# An enigmatic crocodyliform tooth from the bauxites of western Hungary suggests hidden mesoeucrocodylian diversity in the Early Cretaceous European archipelago

**DOI:** 10.7717/peerj.1160

**Published:** 2015-08-13

**Authors:** Attila Ősi, Márton Rabi, László Makádi

**Affiliations:** 1 Department of Paleontology, Eötvös University, Budapest, Hungary; 2 MTA–ELTE Lendület Dinosaur Research Group, Budapest, Hungary; 3 Institute of Geosciences, University of Tübingen, Tübingen, Germany; 4 Geological and Geophysical Institute of Hungary, Budapest, Hungary

**Keywords:** Notosuchia, Paralligatoridae, Early Cretaceous, Alsópere Bauxite Formation, Albian, Hungary

## Abstract

**Background.** The Cretaceous of southern Europe was characterized by an archipelago setting with faunas of mixed composition of endemic, Laurasian and Gondwanan elements. However, little is known about the relative timing of these faunal influences. The Lower Cretaceous of East-Central Europe holds a great promise for understanding the biogeographic history of Cretaceous European biotas because of the former proximity of the area to Gondwana (as part of the Apulian microcontinent). However, East-Central European vertebrates are typically poorly known from this time period. Here, we report on a ziphodont crocodyliform tooth discovered in the Lower Cretaceous (Albian) Alsópere Bauxite Formation of Olaszfalu, western Hungary.

**Methods.** The morphology of the tooth is described and compared with that of other similar Cretaceous crocodyliforms.

**Results.** Based on the triangular, slightly distally curved, constricted and labiolingually flattened crown, the small, subequal-sized true serrations on the carinae mesially and distally, the longitudinal fluting labially, and the extended shelves along the carinae lingually the tooth is most similar to some peirosaurid, non-baurusuchian sebecosuchian, and uruguaysuchid notosuchians. In addition, the paralligatorid Wannchampsus also possesses similar anterior teeth, thus the Hungarian tooth is referred here to Mesoeucrocodylia indet.

**Discussion.** Supposing a notosuchian affinity, this tooth is the earliest occurrence of the group in Europe and one of the earliest in Laurasia. In case of a paralligatorid relationship the Hungarian tooth would represent their first European record, further expanding their cosmopolitan distribution. In any case, the ziphodont tooth from the Albian bauxite deposit of western Hungary belongs to a group still unknown from the Early Cretaceous European archipelago and therefore implies a hidden diversity of crocodyliforms in the area.

## Introduction

During the mineral explorations in the Transdanubian Range of Hungary, Central-East Europe, various bauxite deposits have been discovered and studied in the Bakony Mountains, among others, by Jenö Noszky Jr. and colleagues (Noszky, 1951; Mindszenty, Szöts & Horváth, 1989). In 1950, during fieldwork at the Boszorkány Hill close to the village Olaszfalu, Noszky found a tooth and an unidentified bone fragment in a piece of bauxitic clay. Kretzoi & Noszky (1951) briefly described (but did not figure) the tooth and identified it as crocodilian.

Although Kretzoi & Noszky (1951) did not assign accession number to the specimen in their description, it has been presumed that it was deposited in the collection of the Hungarian Geological Museum of the Hungarian Geological Institute (MÁFI; now Geological and Geophysical Institute of Hungary [MFGI], Department of Geological and Geophysical Collections). The wherabouts of the tooth were unknown until late 2014, when one of us (LM) located it among uncatalogued vertebrate specimens of the collection. The tooth was found in a small box without an inventory number but with a label indicating its identity. Next to it was a walnut-sized piece (and smaller fragments) of bauxite still embedding presumably the same indeterminate bone fragment that was mentioned by Kretzoi & Noszky (1951). The bauxite pieces exhibit cut marks and fit to a fist-sized piece of bauxite housed in the mineralogical collection (inv. no.: MFGI ÁT 5868). This fist-sized rock in turn lacks the cut out portions but has an inventory card that indicates that the “Saurius tooth” was found in it. Thus, it is clear that Noszky found the tooth and the bone fragment in this fist-sized bauxitic clay (MFGI ÁT 5868), and cut out the fossil-containing portions. The remaining piece of rock was catalogued and placed in the mineralogical collection as a bauxite sample, whereas the tooth and the bone were put into the vertebrate collection, where they remained uncatalogued even after the publication of Kretzoi & Noszky (1951). The specimens were catalogued properly only while preparing the present paper under the inventory numbers MFGI V 2015.90.2.1. (tooth) and MFGI V 2015.90.2.2. (bone fragment).

In this paper, we give a new and comparative description of this isolated tooth briefly mentioned by Kretzoi & Noszky (1951) and discuss its taxonomic affinity and paleobiogeographic significance in light of the currently available crocodyliform record.

## Locality, Geological Setting and Age

**Figure 1:**
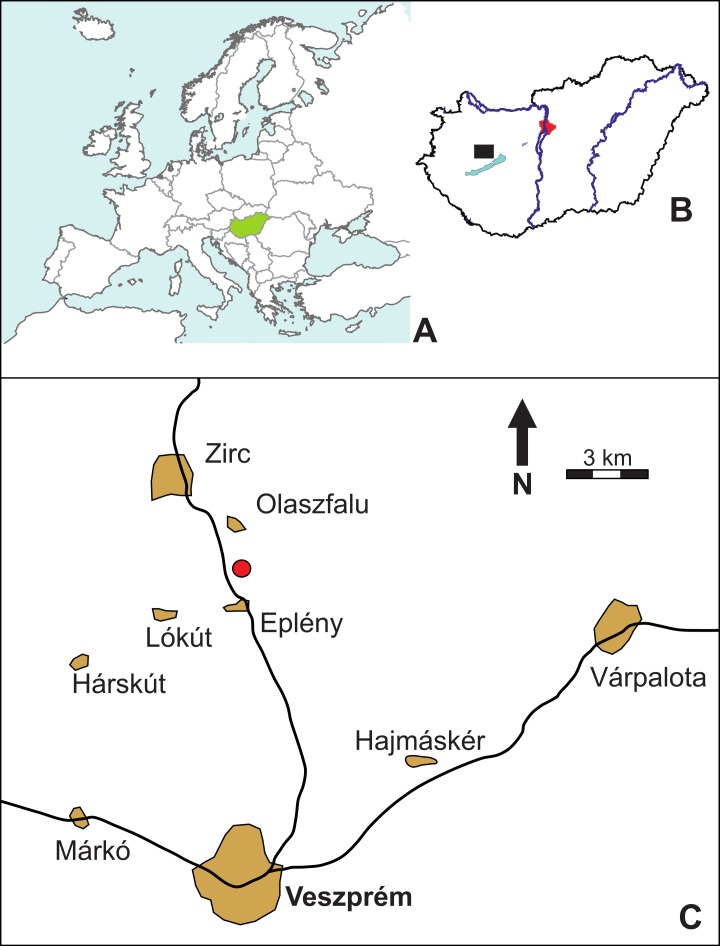
Location map (red circle) of the Mesoeucrocodylia indet. tooth (MFGI V 2015.90.2.1.), found between the villages of Olaszfalu and Eplény in the Bakony Mountains, western Hungary. (A) Hungary in Central Europe. (B) Location of the Olaszfalu area in Hungary. (C) The locality close to the villages of Olaszfalu and Eplény.

The piece of bauxitic clay that contained the tooth together with the small chunk of unidentified bone was collected in a small pit-like depression at the Boszorkány Hill, south of the village of Olaszfalu (Fig. 1), Bakony Mountains, western Hungary (Kretzoi & Noszky, 1951). The specimen came from a fault zone containing the Lower Cretaceous Alsópere Bauxite Formation. The embedding bauxitic rock, “according to thermic analyses, is not bauxite, but a clay consisting of caolinites, but stratigraphically it is equivalent to the Alsópere Bauxite” (Kretzoi & Noszky, 1951). The Alsópere Bauxite Formation occurs in small lenses with a maximum thickness of 5–7 m and was deposited on the karstic surface of the Upper Triassic Dachstein Limestone and in some places on the eroded surface of Liassic limestones. Its lithological features are best represented by the stratigraphic column of the Ot-84 borehole at Olaszfalu (Császár et al., 1993: Fig. 5). The Alsópere Bauxite Formation is a terrestrial deposit mainly built up of allite and kaolinite. It is quite heterogenous containing reddish bauxitic clay and brownish-reddish clayey bauxite.

**Figure 2:**
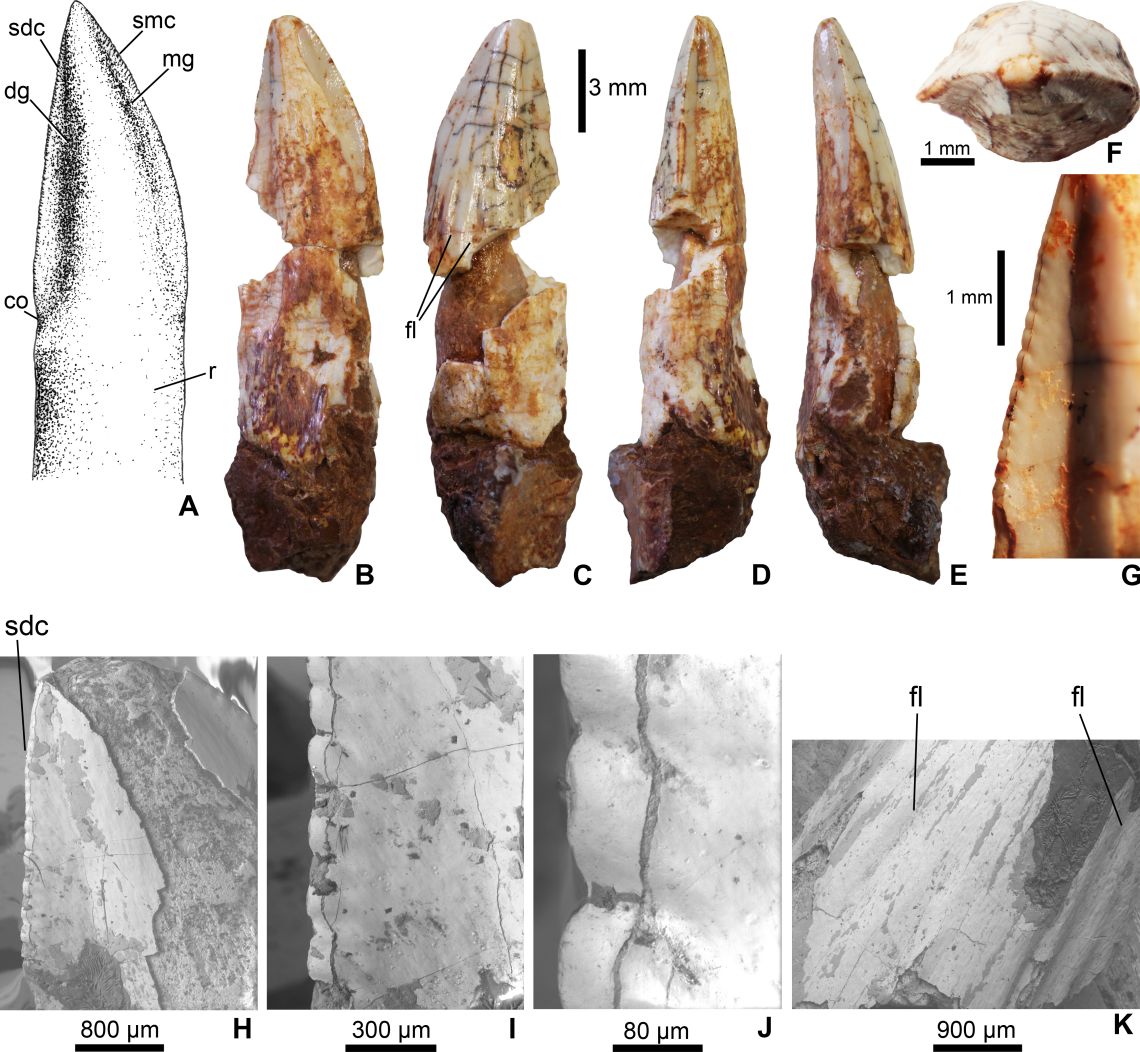
Mesoeucrocodylia indet. crocodyliform tooth (MFGI V 2015.90.2.1.) from the Lower Cretaceous (Lower Albian) Alsópere Bauxite Formation. (A) Reconstruction of the tooth in lingual view. (B) The tooth in lingual; (C) labial; (D) distal; (E) mesial; (F) apical view. (G)–(J), Details of the serrated distal carina. (K) Details of the flutings on the labial side of the tooth. Abbreviations: co, constriction between the crown and root; dg, distal groove; fl, fluting on the enamel surface; mg, mesial groove; sdc, serrated distal carina; smc, serrated mesial carina; r, root.

As is usual with bauxites, the age of the Alsópere Bauxite Formation can only be indirectly established from the age of the underlying and overlying deposits. Although not observable directly at the locality, the youngest underlaying beds are members of the Uppermost Aptian Tata Limestone Formation indicating a younger age for the Alsópere Bauxite. In the surrounding area of the locality, the Alsópere Bauxite Formation is covered by the Tés Clay Formation, representing a transitional unit from terrestrial, paludal to marine sedimentary environments. Based on sporomorphs (Juhász, 1979; Juhász, 1983), foraminifers and ostracods (Császár, 1986), the age of the Tés Clay Formation is Middle–Upper Albian. The stratigraphic record therefore indicates a Lower Albian age for the Alsópere Bauxite Formation (Császár, 1986; Császár et al., 1993; Császár, Fözy & Mizák, 2008).

## Results and Discussion

### Description

#### Orientation

A common characteristic of conical crocodyliform teeth is that they curve somewhat lingually and/or distally. When these teeth are longer than wide (in the horizontal plane) then the greater dimension corresponds to the mesiodistal length and the shorter one to the labiolingual width. Based on these general features, we interpret the slightly concave surface between the two carinae as the lingual and the more convex surface as the mesial side of the crown.

#### Morphology

The tooth (MFGI V 2015.90.2.1.) has a whitish color most probably as a result of oxidation. The central part of the crown missing, but having a pulp cavity completely filled with sediment (Fig. 2). It has a high, apically pointed, triangular, and slightly distally and lingually curved crown. The apicobasal length of the crown is 16 mm, the mesiodistal width is 5 mm and the labiolingual thickness is 3 mm; thus, the crown is slightly labiolingually flattened. The mesial and distal carinae of the crown are preserved only on the apical third and are denticulated (Figs. 2A, 2B and 2H). Following the definitions of Legasa, Buscalioni & Gasparini (1994) and Prasad & Lapparent de Broin (2002), in the case of true ziphodonts the carina is composed of isolated denticles separated by interdenticle grooves. The serration of MFGI V 2015.90.2.1. (Fig. 2G) is closer to the true ziphodont type in having individual denticles on the carinae. The interdenticle grooves of the serrated carinae are quite shallow and slightly curve ventrally towards the central region of crown (at least along the preserved apical part; Fig. 2J). Nevertheless, these denticles are clearly not the marginal prolongation of the enamel ridges as would be expected in a pseudoziphodont tooth. The outer keel of the denticles is rounded (Figs. 2G–2J). Based on the incomplete, preserved part of the carinae the average serration density on both the mesial and distal carinae is 6 denticles per mm. The lingual side of the crown bears a central convexity bordered by a pair of grooves mesially and distally, which in turn support the denticulated carinae. The distal groove is slightly wider mesiodistally than the mesial one (Figs. 2A and 2B). Similar grooves cannot be observed on the labial side of the crown. Labially, at least six shallow, longitudinal flutes occur in the basal part and terminate at the mid-length of the crown (Fig. 2C). The base of the crown is poorly preserved but on the distal side a slight constriction can be observed (Figs. 2A and 2B). The tooth base is still embedded in a piece of bauxitic matrix, but the root is visible both on the lingual and labial sides.

### Comparison and taxonomic assignment

Thecodont teeth with serrated carinae are known in a variety of Mesozoic amniotes including plesiosaurians (e.g., Massare, 1987), basal archosauriforms (e.g., Abler, 1992; Senter, 2003; Beatty & Heckert, 2009), basal pterosaurs (e.g., Ősi, 2011), theropod dinosaurs (e.g., Smith, Vann & Dodson, 2005), and crocodyliforms (e.g., Prasad & Lapparent de Broin, 2002; Andrade et al., 2010; Marinho et al., 2013; Rabi & Sebők, in press; Martin, in press).

#### Plesiosaurs

Among plesiosaurs, some pliosaurs have teeth with ziphodont carinae and slightly flattened crown, but the longitudinal fluting or the lingual grooves along the carinae do not appear in these forms. Their teeth are usually conical and elongated frequently with coarse striations and without constrictions between the crown and the root (e.g., Massare, 1987; Sasson, Noè & Benton, 2012).

#### Spinosaurid theropods

The subcircular cross-section and longitudinal fluting along the crown of spinosaurids are comparable to the tooth from Hungary in some aspects. Spinosaurid teeth, however, have no constriction between the crown and the root, and teeth are usually much larger and more robust (see e.g., Canudo et al., 2008). The enamel of spinosaurids is distinctly fluted by relatively wide grooves extending the along the entire height of the crown both labially and lingually in most species (but see *Baryonyx*, Charig & Milner, 1986; Charig & Milner, 1997; Buffetaut, 2007). These flutes are much wider (the ridges between the flutes are essentially crest-like, e.g., see Kellner, 1996; Buffetaut, 2008; Buffetaut, 2013) than those seen in the Hungarian tooth. No spinosaurid teeth have the shelf-like, lingual grooves along the carinae mesially and distally as seen in MFGI V 2015.90.2.1.

#### Protosuchians

Protosuchian crocodyliforms show a great variety of dentition including some ziphodont forms. Among the two species of the Early Cretaceous *Sichuanosuchus* (Peng, 1996; Wu, Sues & Dong, 1997), only *S*. *huidongensis* possesses teeth with serrated carinae. Here, both the premaxillary and maxillary (including the posterior ones) teeth are finely serrated, and the latter teeth are compressed labiolingually. Neither the longitudinal flutes, nor the lingual grooves along the mesial and distal carina are present in this basal form (Peng, 1996). Dental features similar to those of *S. huidongensis* have been described for the Upper Jurassic *Hsisosuchus chungkingensis* (Young & Chow, 1953).

#### Metriorhynchids

Among metriorhynchid thalattosuchians, the cosmopolitan *Dakosaurus* (Mason, 1869; Gasparini, Pol & Spalletti, 2006), and *Geosaurus* (Andrade et al., 2010) possess tooth morphology broadly similar to that seen in MFGI V 2015.90.2.1. Posterior maxillary and dentary teeth of *Dakosaurus* are robust, conical, labiolingually compressed, and mesiodistally serrated, but lack the grooves mesially and distally along the lingual side of the carinae (Gasparini, Pol & Spalletti, 2006). The teeth of *Geosaurus* from the Late Jurassic of Germany are more compressed labiolingually and they are much more like an isosceles triangle with almost straight mesiodistal carinae (Andrade et al., 2010) in contrast with the slightly curved carinae of MFGI V 2015.90.2.1. Besides morphological differences, a further suggestive argument is that the stratigraphic position of the specimen in the unambiguously terrestrial Alsópere Bauxite Formation (Császár, 1986) makes a marine crocodyliform identity highly unlikely.

#### Baurusuchids

In *Baurusuchus* (MPMA-62-0001-02; Carvalho, Campos & Nobre, 2005; Vasconcellos & Carvalho, 2007; Riff & Kellner, 2001, Ösi A. pers. obs.) and *Campinasuchus* (Carvalho et al., 2011), the hypertrophied teeth are robust, subcircular in cross-section, and the carinae on the mesial and distal edges are serrated with marked denticles. Tooth crowns lack the longitudinal grooves mesially and distally on the lingual side and the longitudinal fluting labially, and the crowns are not or very slightly constricted. The two poorly preserved teeth of *Wargosuchus* (Martinelli & Pais, 2008) show similar morphology as well. The teeth of *Pissarrachampsa* are generally similar to those of other baurusuchids, but the maxillary and posterior dentary tooth crowns are laterally strongly compressed. However, neither mesiodistally positioned longitudinal grooves mesially and distally on the lingual side, nor labial longitudinal fluting are present (Montefeltro, Larsson & Langer, 2011). In *Pabwehshi*, the anterior teeth are similar to those of other baurusuchids, but all the teeth bear longitudinal striae (Wilson, Malkani & Gingerich, 2001) making them different from MFGI V 2015.90.2.1. The teeth of *Gondwanasuchus* (Marinho et al., 2013) are similar to the Hungarian specimen in having labiolingually compressed, serrated crowns. They bear five or six deep and wide longitudinal flutes that converge apically and are separated by ridges. Similar longitudinal fluting is present on MFGI V 2015.90.2.1. as well, though these flutes are more shallow and are not present in the apical half of the crown. As in many baurusuchids, the distal carina of the strongly curved teeth of *Gondwanasuchus* is concave in contrast to the slightly convex carina present in the Hungarian specimen. In conclusion, the teeth of baurusuchids are generally similar to MFGI V 2015.90.2.1. but the latter shows a combination of morphological characters that clearly distinguishes it from the teeth of these genera.

#### Peirosaurids

Peirosaurids show more diverse tooth crown morphology than baurusuchids. The premaxillary, the hypertrophied maxillary, and the dentary teeth of *Montealtosuchus*(MPMA-16-0007-04; Carvalho, Vasconcellos & Tavares, 2007) are basically similar to the Hungarian specimen in having an oval cross-section, slightly convex, finely serrated carinae and slightly constricted crown, but they lack the grooves mesially and distally on the lingual side and the longitudinal fluting labially. *Pepesuchus* (Campos et al., 2011) differs from MFGI V 2015.90.2.1. in having triangular teeth with striated external surfaces and well-marked longitudinal lines on the crowns, as well as in the carinae lacking serrations. The teeth of *Uberabasuchus* (Carvalho, Ribeiro & Avilla, 2004) are much more like those of baurusuchids in having massive conical teeth, with serrations posteriorly only. *Barcinosuchus* possesses teeth on which the serrations are quite similar to those on the Hungarian tooth (Leardi & Pol, 2009: Fig. 3G), though the presence or absence of serration on the carinae varies among the teeth. In *Lomasuchus* (Gasparini, Chiappe & Fernandez, 1991), the anterior, more pointed teeth are serrated but on the other hand they exhibit a flat lingual and convex labial surface without fluting or lingual grooves mesially and distally, in contrast to MFGI V 2015.90.2.1. The pointed, hypertrophied and serrated teeth of *Hamadasuchus rebouli* from the Albian–Cenomanian Kem Kem beds (Larsson & Sues, 2007: Fig. 3) are similar to MFGI V 2015.90.2.1. in having similar longitudinal fluting labially, but these teeth are not as compressed labiolingually and lack the lingually developed grooves mesially and distally along the serrated cutting margin.

#### Mahajangasuchids

This clade, defined by Sereno & Larsson (2009), comprises two Late Cretaceous bizarre forms, *Mahajangasuchus insignis* and *Kaprosuchus saharicus*, of which the former species has labiolingually compressed tooth crowns with serrated carinae. These teeth differ from the Hungarian tooth in being extremly robust in cross-section and without fluting or lingual grooves along the carinae (Turner & Buckley, 2008). *Kaprosuchus* possesses labiolingually compressed teeth with smooth mesial and distal carinae (Sereno & Larsson, 2009).

#### Trematochampsids

Regarding trematochampsids, the teeth of *Trematochampsa* from the Lower Senonian of Niger (Buffetaut, 1976) are comparable with the tooth from Olaszfalu. Teeth of this genus are massive but some of them are labiolingually compressed (Buffetaut, 1976: pl. 6, Fig. 3). However, these differ from the Hungarian specimen in having longitudinal enamel striae and in the absence of the lingually developed grooves mesially and distally along the serrated cutting margin.

#### Non-baurusuchid sebecosuchians

Among these forms the teeth of *Doratodon carcharidens* from the Santonian of Hungary (Rabi & Sebők, in press: Fig. 4) and *Doratodon ibericus* from the Campanian of Spain (Company et al., 2005) are most similar to MFGI V 2015.90.2.1. The teeth of these species also possess slightly concave grooves towards the mesial and distal carinae, and in *D. ibericus* the longitudinal flutes occur labially as well. Serration of the carinae of both species is, however, more pronounced, and the crown of *D. carcharidens* is more constricted basally than that seen in the Olaszfalu specimen. Among sebecids, the teeth of *Sebecus* are similar in having flattened, pointed, triangular tooth crowns with serrations (Colbert, 1946: Fig. 21) but the labial fluting and the lingual grooves along the carinae are not present on the teeth. The same features can be observed in the teeth of *Iberosuchus* (Ortega, Buscalioni & Gasparini, 1996), though they are more distally curved than the Hungarian specimen. *Ilchunaia* (Gasparini, 1972) differs from MFGI V 2015.90.2.1. in having two additional carinae on the crown. Besides these forms, *Sahitisuchus* possesses ziphodont, straight or posteriorly curved teeth with pointed and labiolingually compressed crowns (see e.g., the 4th, left mandibular tooth in Kellner, Pinheiro & Campos, 2014: Fig. 6), but labial fluting is not present in the teeth.

#### Uruguaysuchids

Among uruguaysuchids the teeth of *Araripesuchus wegeneri* show features similar to the tooth from Olaszfalu. Enlarged teeth, though proportionally not as high as the Hungarian tooth, are present in this species in the anterior parts of the dentary and maxilla. The teeth are labiolingually flattened and pointed, and have lingual grooves (“trough” of Sereno & Larsson, 2009: 51) along the carinae mesiodistally. Dentary teeth have finely denticulate margins and fluting occurs on the lingual surface of the enlarged, fourth premaxillary tooth. These flutes, however, appear to be more dense in the teeth of *A*. *wegeneri*Sereno & Larsson, 2009: Fig. 19A) than in the Hungarian specimen.

#### Planocraniids

Among neosuchians some planocraniids possess ziphodont dentition (Brochu, 2013) but similar to the condition in most notosuchians they also lack the labial fluting and lingual grooves along the carinae.

#### Paralligatorids

This recently revised clade of non-eusuchian neosuchian (Montefeltro et al., 2013) or possibly eusuchian (Turner, 2015) crocodilians contains at least one species with a tooth morphology similar to the Hungarian specimen. *Wannchampsus kirpachi* from the Early Cretaceous of North America (Adams, 2014) also possesses labiolingually slightly flattened, ziphodont teeth with narrow, longitudinal fluting on the labial side, constricted crown, and lingual grooves along the carinae mesiodistally. These teeth (isolated but associated with the type material) of *W. kirpachi* differ from the Hungarian tooth in having only modestly compressed crowns labiolingually and strong carinae with denticles (Adams, 2014: Fig. 9). In other paralligatorids, this tooth morphology is not present. Only the oldest member of the group, *Batrachomimus pastosbonensis* (Montefeltro et al., 2013) is comparable. It possesses non-ziphodont teeth with longitudinal fluting along the whole upper tooth row, but these flutes are much finer and more abundant than in the Hungarian specimen (F Montefeltro, pers. comm., 2015).

#### Other mesoeucrocodylians

Some other crocodyliforms possess teeth with generally similar morphology as well. An isolated tooth referred to Notosuchia indet. from Coniacian–Santonian beds of Italy (Dalla Vecchia & Cau, 2011) is similar to the Hungarian specimen in having labiolingually flattened, pointed, triangular crown with ziphodont carinae. However, the slightly concave distal carina, the lingually shifting mesial carina, the lack of fluting on the crown surface and the marked constriction below the distal carina clearly distinguish these two types of teeth from each other. Hypertrophied teeth of the atoposaurid *Theriosuchus*, for example, have striae on the sides that are inclined and terminate in the carinae resulting in pseudoziphodont morphology (Martin et al., 2014).

To sum up, we can conclude that the tooth from the Albian Alsópere Bauxite Formation, western Hungary does not bear diagnostic features unambiguously referring it to any certain clade of crocodyliforms, but is most similar to the ziphodont teeth of some peirosaurid, non-baurusuchian sebecosuchian, and uruguaysuchid notosuchians (sensu Sereno et al., 2001; Pol et al., 2014). Among peirosaurids, the hypertrophied, labiolingually slightly flattened teeth of the North African Albian *Hamadasuchus* are the most similar to the specimen described here in having labial fluting and serrated carinae. Among sebecosuchians, the European Late Cretaceous *Doratodon ibericus* shows the greatest similarity with the Early Cretaceous Hungarian tooth. The enlarged teeth of *Araripesuchus wegeneri* are also similar in various aspects.

Besides notosuchians, it closely resembles the teeth of the paralligatorid *Wannchampsus kirpachi*. On the basis of these comparative results, we refer the tooth from Olaszfalu to Mesoeucrocodylia indet., until more complete material helps to clarify its precise taxonomic assignment.

## Paleobiogeographic Inferences

Since the tooth from Olaszfalu either represents a notosuchian (sensu Sereno et al., 2001; Pol et al., 2014) or a paralligatorid (Adams, 2014; Turner, 2015) neosuchian crocodyliform, two paleobiogeographic scenarios can be outlined.

In case of a notosuchian affinity, this tooth represents the earliest indication of notosuchian crocodyliforms on European landmasses. Previously, the remains of this clade were known only from Cenomanian to Eocene deposits in different regions of the European archipelago: *Hamadasuchus*-like teeth from the Cenomanian of France (Vullo et al., 2005; Vullo & Néraudeau, 2008), a Coniacian–Santonian aged isolated tooth referred to Notosuchia from Italy (Dalla Vecchia & Cau, 2011), remains of the notosuchian *Doratodon carcharidens* from the Santonian of Iharkút, Hungary (Rabi & Sebők, in press) and from the Lower Campanian of Muthmannsdorf, Austria (Buffetaut, 1979), and *Doratodon ibericus* from the Campanian of Spain (Company et al., 2005). *Doratodon* has been reported from the Lower Maastrichitian of Romania as well (Grigorescu et al., 1999). Finally, a number of fragmentary remains from the Paleogene of Europe are referred mainly to sebecosuchians (Buffetaut, 1980; Buffetaut, 1986; Ortega, Buscalioni & Gasparini, 1996; Martin, in press, and references therein). With a notosuchian affinity, the Hungarian tooth would date back the European occurrence of the otherwise primarily Gondwanan group to the Early Cretaceous (Early Albian). The other non-Gondwanan notosuchian is *Chimaerasuchus paradoxus* from Aptian–Albian deposits of China (Wu, Sues & Sun, 1995). Though the material is fragmentary, bearing numerous highly apomorphic features, most phylogenetic analyses have found *Chimaerasuchus* being nested well within Notosuchia (Pol et al., 2014). The phylogeny of Pol et al. (2014) dates the origin of basal notosuchians to the Early Jurassic and infers an almost 60 My long ghost lineage (i.e., the first half of notosuchian evolution; Pol et al., 2014: Fig. 47). The hypothesis of Pol et al. (2014) argues for a more complex biogeographic history of the group, and therefore their Laurasian temporal distribution is perhaps well underestimated. The Hungarian specimen may suggest that notosuchian crocodyliforms existed already in the Early Albian in the southern part of the European archipelago. If the tooth from Olaszfalu is from a notosuchian, then, along with Santonian neobatrachian anurans (Szentesi & Venczel, 2010), Coniacian–Santonian notosuchians (Dalla Vecchia & Cau, 2011; Rabi & Sebők, in press), Santonian bothremydid turtles (Rabi, Tong & Botfalvai, 2012; Rabi, Vremir & Tong, 2013), and Albian and Santonian abelisaurids (Accarie et al., 1995; Ősi & Buffetaut, 2011), all clades of Gondwanan origin, it would suggest that faunal links between the European archipelago and Africa might have existed during most of the Cretaceous (Csiki-Sava et al., 2015; Rabi & Sebők, in press); contra (Ezcurra & Agnolín, 2012).

Based on the similar dental characters seen in the North American *Wannchampsus kirpachi*, a paralligatorid affinity is also plausible, though the exact tooth morphology of *W. kirpachi* is actually not present in other paralligatorid forms. In case of a paralligatorid affinity, the Hungarian tooth would represent the first European record of the group further expanding their cosmopolitan distribution.

Either a notosuchian, or a paralligatorid, the tooth from the Albian bauxite deposit of western Hungary represents a group still unknown from the Early Cretaceous European archipelago, and therefore implies a hidden diversity of crocodyliforms in the area. The currently known record of late Early Cretaceous (Barremian–Albian) European crocodyliforms includes goniopholidids (Andrade et al., 2011), possible hylaeochampsids (Buscalioni et al., 2011), atoposaurids (Brinkmann, 1992; Schwarz & Salisbury, 2005), and bernissartiids (Buffetaut & Ford, 1979; Buscalioni & Sanz, 1990; Sweetman, Pedreira-Segade & Vidovic, 2015), none of them having ziphodont teeth. The ziphodont tooth from Olaszfalu from a certainly terrestrial deposit of the Transdanubian Range (Apulian microplate, Csontos & Vörös, 2004) may suggest the existence of terrestrial crocodyliforms in the European archipelago. Hopefully, future discoveries will reveal the affinities of this peculiar taxon, and help to specify the composition of European Early Cretaceous crocodyliform diversity.

## Conclusions

Rabi & Sebők (in press) recently noted that there is no sign of true ziphodont crocodyliforms in the Early Cretaceous of Europe. The tooth (MFGI V 2015.90.2.1.) from the Albian Alsópere Bauxite Formation, western Hungary, however, is ziphodont and closely resembles that of some peirosaurid, non-baurusuchian sebecosuchian, and uruguaysuchid crocodyliforms—all of which have been united into a single clade, Notosuchia (Sereno et al., 2001; Pol et al., 2014). In case of notosuchian affinity this tooth would represent the earliest indication of the clade in Europe. Besides notosuchians, the paralligatorid *Wannchampsus kirpachi* possesses similar dental features to the tooh presented here. If the Hungarian specimen is from a paralligatorid, then it would be the first occurrence of the group in the European archipelago. On the basis of these comparative results, we refer the tooth from Olaszfalu to Mesoeucrocodylia indet., until more complete material helps to clarify its precise taxonomic assignment. The tooth from the Albian of western Hungary certainly represents a group still unknown from the European Lower Cretaceous, and therefore adds to the diversity of Early Cretaceous crocodyliforms in the area.

## Institutional abbreviations

MFGI: Magyar Földtani és Geofizikai Intézet, Budapest, Hungary
MGUV: Museo del Departamento de Geología, Universidad de Valencia, Burjassot, Spain
MPMA: Museu de Paleontologia de Monte Alto, Monte Alto, Brazil
